# Mannose 6-Phosphate Receptor and Sortilin Mediated Endocytosis of α-Galactosidase A in Kidney Endothelial Cells

**DOI:** 10.1371/journal.pone.0039975

**Published:** 2012-06-29

**Authors:** Thaneas Prabakaran, Rikke Nielsen, Simon C. Satchell, Peter W. Mathieson, Ulla Feldt-Rasmussen, Søren S. Sørensen, Erik I. Christensen

**Affiliations:** 1 Department of Biomedicine, Aarhus University, Aarhus, Denmark; 2 Academic Renal Unit, University of Bristol, Bristol, United Kingdom; 3 Department of Medical Endocrinology, Rigshospitalet, Copenhagen, Denmark; 4 Department of Nephrology P, Rigshospitalet, Copenhagen, Denmark; Biological Research Centre of the Hungarian Academy of Sciences, Hungary

## Abstract

Prominent vasculopathy in Fabry disease patients is caused by excessive intracellular accumulation of globotriaosylceramide (GL-3) throughout the vascular endothelial cells causing progressive cerebrovascular, cardiac and renal impairments. The vascular lesions lead to myocardial ischemia, atherogenesis, stroke, aneurysm, thrombosis, and nephropathy. Hence, injury to the endothelial cells in the kidney is a key mechanism in human glomerular disease and endothelial cell repair is an important therapeutic target. We investigated the mechanism of uptake of α-galactosidase A (α-Gal A) in renal endothelial cells, in order to clarify if the recombinant enzyme is targeted to the lysosomes via the universal mannose 6-phosphate receptor (M6PR) and possibly other receptors. Immunohistochemical localization of infused recombinant α-Gal A in a renal biopsy from a classic Fabry disease patient showed that recombinant protein localize in the endothelial cells of the kidney. Affinity purification studies using α-Gal A resins identified M6PR and sortilin as α-Gal A receptors in cultured glomerular endothelial cells. Immunohistochemical analyses of normal human kidney with anti-sortilin and anti-M6PR showed that sortilin and M6PR were expressed in the endothelium of smaller and larger vessels. Uptake studies in cultured glomerular endothelial cells of α-Gal A labeled with fluorescence and ^125^I showed by inhibition with RAP and M6P that sortilin and M6PR mediated uptake of α-Gal A. Biacore studies revealed that α-Gal A binds to human M6PR with very high affinity, but M6PR also binds to sortilin in a way that prevents α-Gal A binding to sortilin. Taken together, our data provide evidence that sortilin is a new α-Gal A receptor expressed in renal endothelial cells and that this receptor together with the M6PR is able to internalize circulating α-Gal A during enzyme replacement therapy in patients with Fabry disease.

## Introduction

Fabry-Anderson disease is an X-linked lysosomal storage disorder caused by deficient activity of the lysosomal enzyme α-galactosidase A (α-Gal A) [1], [2]. The enzyme plays a critical role in glycosphingolipid metabolism and deficiency causes accumulation of neutral glycosphingolipids, mainly globotriaosylceramide (GL-3) in the endothelium, epithelial and smooth muscle cells of different organs. The progressive GL-3 accumulations in the vascular endothelial cells (ECs) of the skin, eye, heart, kidney, brain, and peripheral nervous system accounts for the prominent vasculopathy leading to progressive cerebrovascular, cardiac and renal impairments and premature death [1].

End-stage renal failure occurs in some of the patients with Fabry disease in their third to fifth decade of life, when the lysosomal GL-3 accumulation becomes irreversible [3], [4] and patients are in need of dialysis or renal transplantation [4], [5].

Enzyme replacement therapy (ERT) results in significant clearance of GL-3 deposits throughout the vascular endothelium [6], [7]. ERT reduces the GL-3 deposits in the GECs and other renal ECs [1], [7], [8], [9], thereby stabilizing kidney function in patients with Fabry nephropathy, if instituted sufficiently early [10]. Therapeutic effectiveness of ERT with α-Gal A is dependent on endocytosis by recognition of mannose 6-phosphate (M6P) residues on the enzyme by the widely distributed cation-independent M6P receptor/insulin-like growth II receptor [11], [12], [13]. Although ERT has been demonstrated to be efficacious in treatment for Fabry disease, there are many unanswered questions related to the ability of recombinant proteins to target key tissues. Hence, the effectiveness of treatment with ERT in Fabry disease needs further clarification by investigating more cell models of different tissues that are prominently affected in Fabry disease. Our previous studies have already shown that the uptake of α-Gal A in proximal tubule cells is mainly mediated by megalin, whereas in podocytes the uptake is mediated by M6PR, megalin, and sortilin [14], [15].

In this study, we have identified two receptors, M6PR and sortilin, as α-Gal A binding proteins important for the delivery of α-Gal A to ECs in the human kidney. Thus, our data indicate that circulating α-Gal A may be taken up by renal ECs by a M6PR- and sortilin- mediated pathway during ERT in Fabry disease patients. These data can in term be applied to other ECs in other parts of the body.

**Figure 1:**
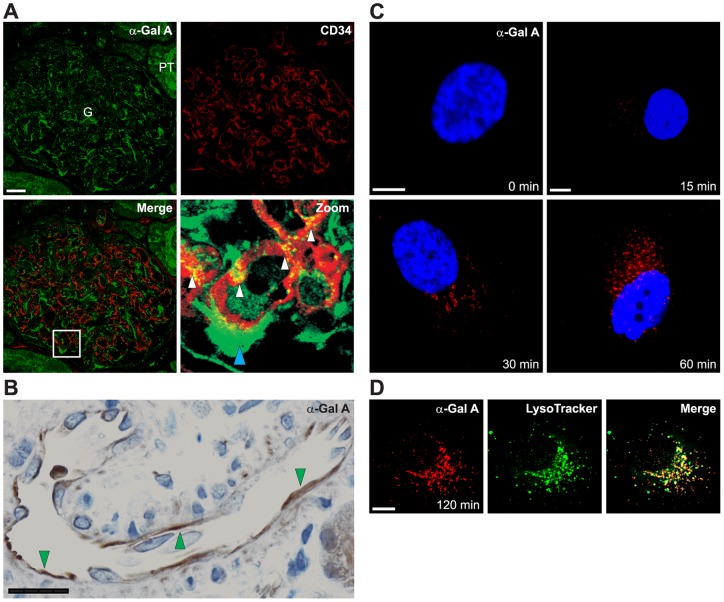
Uptake of α-Gal A by human kidney ECs. (A) Dual immunofluorescence staining showing α-Gal A in the glomerulus (G) in a renal biopsy from a Fabry patient who was infused with α-Gal A 2 h before the biopsy. Localization of α-Gal A is observed in the GECs as indicated by co-localization with CD34 (endothelial cell surface marker). Merged high power view demonstrates that α-Gal A is seen in GECs (white arrowheads) and in podocyte (blue arrowhead) in a human glomerulus from a Fabry disease patient. Labeling for α-Gal A is also seen in the proximal tubule (PT). Scale bar, 25 µm. (B) Peroxidase staining showing localization of α-Gal A (green arrowheads) in larger vessel ECs in a renal biopsy from a Fabry patient who was infused with α-Gal A 2 h before the biopsy. Scale bar, 25 µm. (C) Uptake of Alexa-Fluor 546-labeled α-Gal A (red) in cultured human GECs as a function of time at 37°C. At the indicated times, the cells were fixed and analyzed by confocal microscopy. Scale bar, 5 µm. (D) Co-localization (yellow) of α-Gal A (red) and lysosomes (green). A merged image is shown. Scale bar, 5 µm.

## Materials and Methods

### Antibodies and proteins

Recombinant α-Gal A (agalsidase beta, Fabrazyme) and affinity-purified rabbit polyclonal anti-human α-Gal A were provided by Genzyme Corp. (Framingham, MA, USA). Recombinant receptor-associated protein (RAP) was provided by Novo Nordisk A/S (Bagsværd, DK). Affinity purified rabbit polyclonal anti-sortilin was generated against the ectodomain of sortilin, as previously described [16]. Polyclonal rabbit anti-IGF-II/M6PR 3637 was a kind gift from Dr. W. Kiess, Hospital for Children and Adolescence, University of Leipzig, Leipzig, DE. The mannose 6-phosphate (M6P; M3655) and anti-IGF-II receptor (HPA011332) were purchased from Sigma Aldrich (Saint Louis, MO, USA). Recombinant human M6PR (6418-GR) containing the residues Gln^41^-Asp^1365^ was purchased from R&D Systems. Affinity purified soluble human sortilin was a kind gift from Dr. Claus Munck Petersen, Department of Biomedicine, Aarhus University, Aarhus, DK. Polyclonal rabbit anti-sortilin (ab16640) was purchased from Abcam. Monoclonal mouse anti-human CD34, mouse anti-human platelet endothelial cell adhesion molecule (PECAM-1), and peroxidase-conjugated secondary antibodies were all purchased from DAKO A/S (Glostrup, DK). Fluorescence-conjugated secondary antibodies were purchased from Molecular Probes (Eugene, OR, USA). Controls for nonspecific binding were performed with nonspecific rabbit or mouse IgG from DAKO.

**Figure 2:**
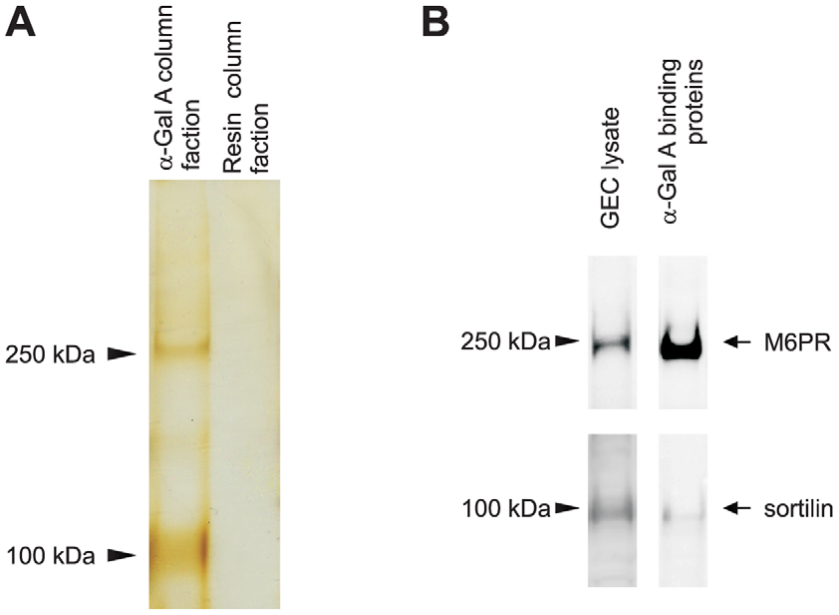
M6PR and sortilin bind specifically to α-Gal A in GECs. (A) Affinity chromatography indicate the migration of the different α-Gal A binding proteins (arrowheads). (B) Western blot analysis demonstrated that the two bands were M6PR and sortilin. Lysate from human GECs culture was used as a positive control.

### Biopsy preparation

A kidney biopsy was obtained from a male Fabry patient, 37 years of age, 2 h after enzyme replacement infusion, 0.2 mg/kg body wt recombinant α-Gal A. Oral consent to biopsy was obtained from the patient according to local regulations. The biopsy was fixed and routine embedded in paraffin. Human tissue sections were also obtained from paraffin-embedded normal human kidneys (autopsy materials).

**Figure 3:**
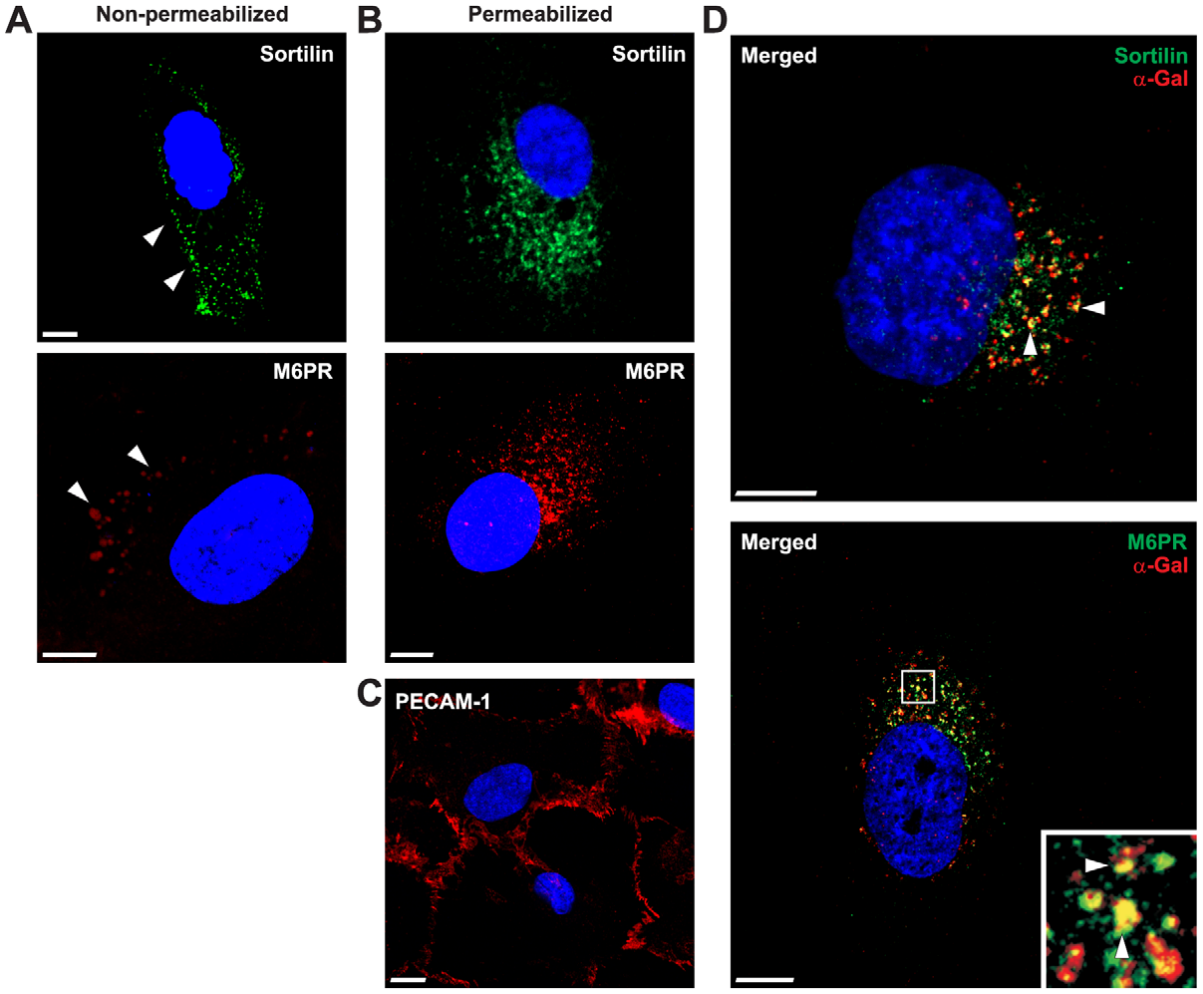
Immunofluorescent demonstration of sortilin, M6PR and PECAM-1 in cultured human GECs. (A) Non-permeabilized GECs showing cell surface labeling of sortilin and M6PR. White arrowheads indicate potential cell surface labeling of the different receptors. (B) Permeabilized GECs showing intracellular labeling for both M6PR and sortilin. (C) Demonstration of the specific endothelial cell marker PECAM-1 in permeabilized GECs. (D) Endocytosed recombinant α-Gal A (red) co-localizes with both sortilin (green) and M6PR (green) in intracellular compartments after 60 min. A high-power view of the co-localization of M6PR with recombinant α-Gal A is shown, yellow color indicated with white arrowheads demonstrates co-localization. Controls were incubated with serum and detected with same secondary antibodies (data not shown). Nuclei (blue) were stained with DAPI. Scale bars, 5 µm.

### Immunohistochemistry

For immunoperoxidase and immunofluorescence staining, 2 μm tissue paraffin sections were cut and prepared as previously described [17]. Immunoperoxidase staining was performed as previously described [17]; briefly sections were incubated with primary antibodies followed by incubation with HRP-conjugated secondary antibody. Peroxidase labeling was visualized by incubation with diaminobenzidine and 0.03% H_2_O_2_ for 10 min and sections counterstained with Meier's hematoxylin. Sections were examined in a Leica DMR microscope equipped with a Leica DFC320 camera. Images were transferred by a Leica TFC Twain 6.1.0 program and processed using Adobe Photoshop 8.0.

**Figure 4:**
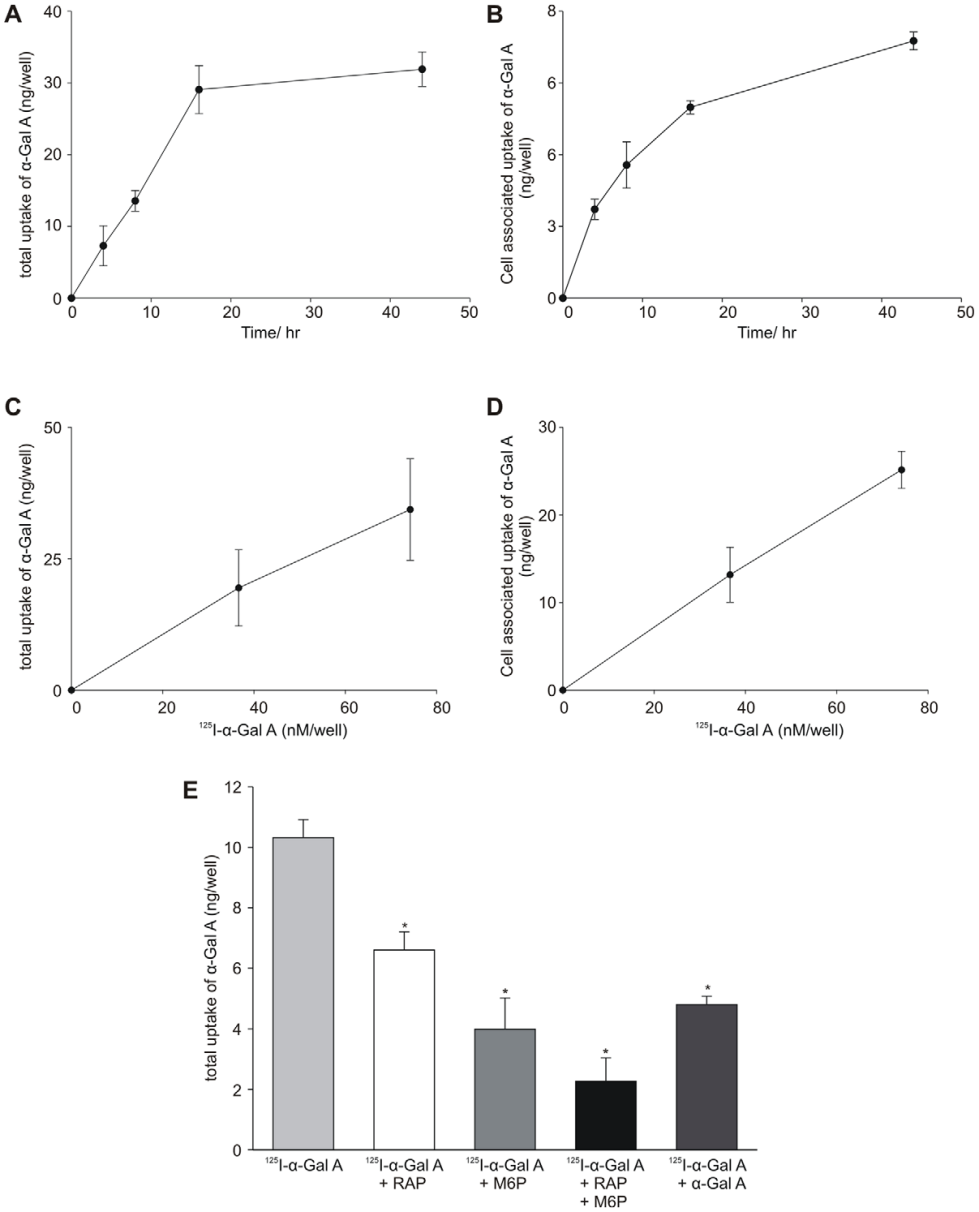
Uptake of ^125^I-labeled α-Gal A by human GECs. (A and B) Human GECs were incubated with ^125^I-α-Gal A for different times at 37°C showing both total and cell-associated α-Gal A uptake. (C and D) Human GECs were incubated with different concentrations of ^125^I-α-Gal A for 12 h at 37°C showing both total and cell-associated α-Gal A uptake. (E) Uptake of ^125^I-α-Gal A and inhibition with M6P, RAP, a combination of both M6P and RAP, and α-Gal A. Uptake was assayed as described in the method section. Each point represents a mean of triplicates with standard deviations. Addition of inhibitors show significant (*) reductions (P<0.002) in the uptake of α-Gal A after 12 h.

For double immunofluorescence staining, sections were incubated with primary mouse monoclonal and rabbit polyclonal antibodies followed by Alexa Fluor conjugated anti-mouse and anti-rabbit IgG. Staining was analyzed by confocal laser scanning microscope (LSM510; Carl Zeiss, DE). Controls; sections were incubated without primary antibodies or with nonspecific IgG.

### Cells

The human glomerular endothelial cells (GECs) conditionally immortalized by introducing temperature-sensitive SV40-T antigen by transfection has previously been characterized in detail [18]. The GECs were maintained in the endothelial growth medium 2 microvascular (EGM2-MV; LONZA, BE) containing foetal calf serum (5%) and growth factors as supplied, at 33°C in 5% CO_2_. GECs differentiation was induced under nonpermissive conditions by thermo shifting the cells to 37°C for 5–7 days. The media was renewed every second day, and cells were split at confluence approximately once a week using a trypsin-EDTA solution (LONZA). Experiments were carried out with confluent monolayers of cells cultured in 24-well plates (Nagle Nunc International, Hereford, UK) with or without cover slips for uptake studies, and in 75 cm^2^ culture flasks (Corning Incorporated, Corning, NY, USA) for the affinity purification experiment.

**Figure 5:**
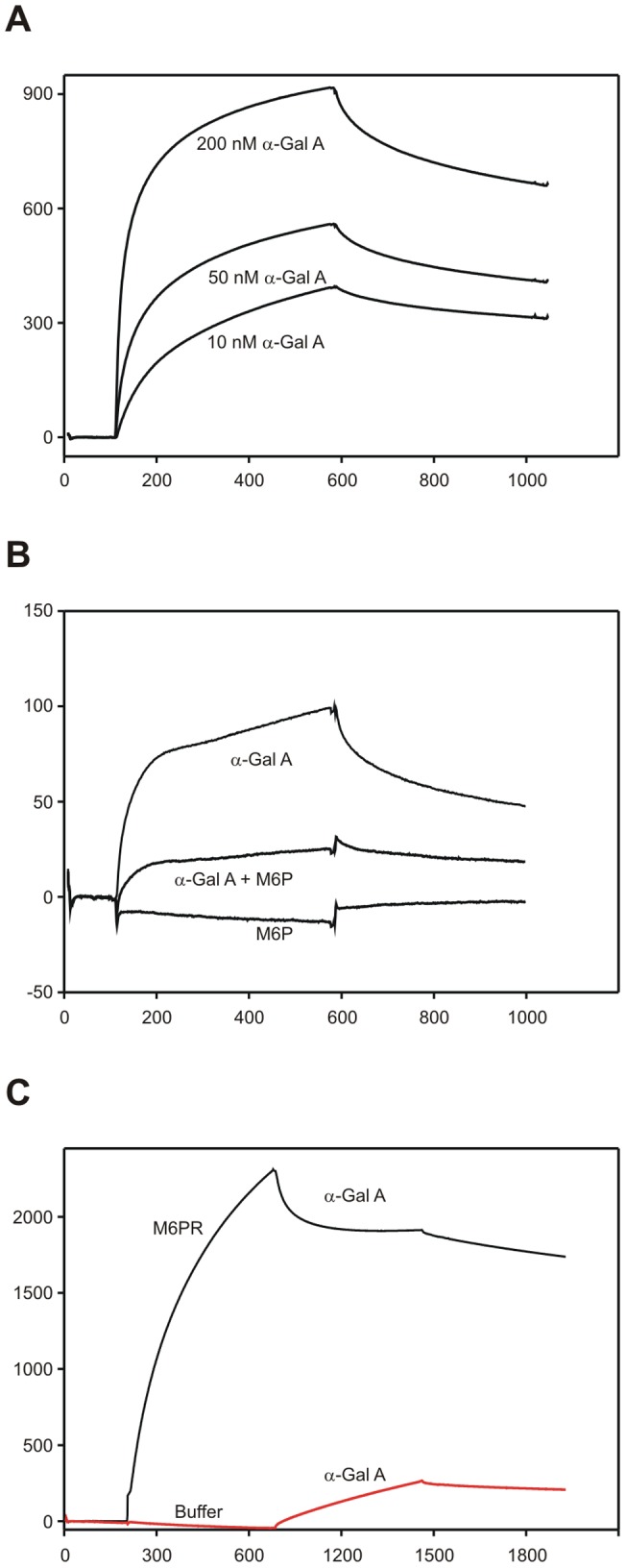
Binding of α-Gal A by M6PR and sortilin. The purified ectodomains of M6PR and sortilin were immobilized on BIAcore chips. (A) SPR analysis of α-Gal A binding to purified human M6PR. (B) Binding of 50 nM α-Gal A to M6PR in the presence or absence of 50 µM M6P. (C) Inhibition of α-Gal A binding to sortilin by M6PR. Sortilin was saturated with M6PR prior to injection of α-Gal A. For comparison, sortilin was saturated with flow buffer prior to injection of α-Gal A.

### Immunofluorescence microscopy

#### Uptake of Alexa Fluor 546-labeled α-Gal A in cultured human GECs

Recombinant α-Gal A was labeled with Alexa Fluor 546 according to the instructions of the manufacturer (Molecular Probes). Coverslips with GECs were incubated with 400 nM Alexa Fluor 546-labeled α-Gal A at 37°C at indicated times, fixed with 4% paraformaldehyde for 10 min at room temperature and counterstained with DAPI (Molecular Probes). For co-localization with lysosomal compartments GECs were incubated with Alexa Fluor 546-labeled α-Gal A and LysoTracker Green DND-26 (L-7526; Molecular Probes) for 2 h.

**Figure 6:**
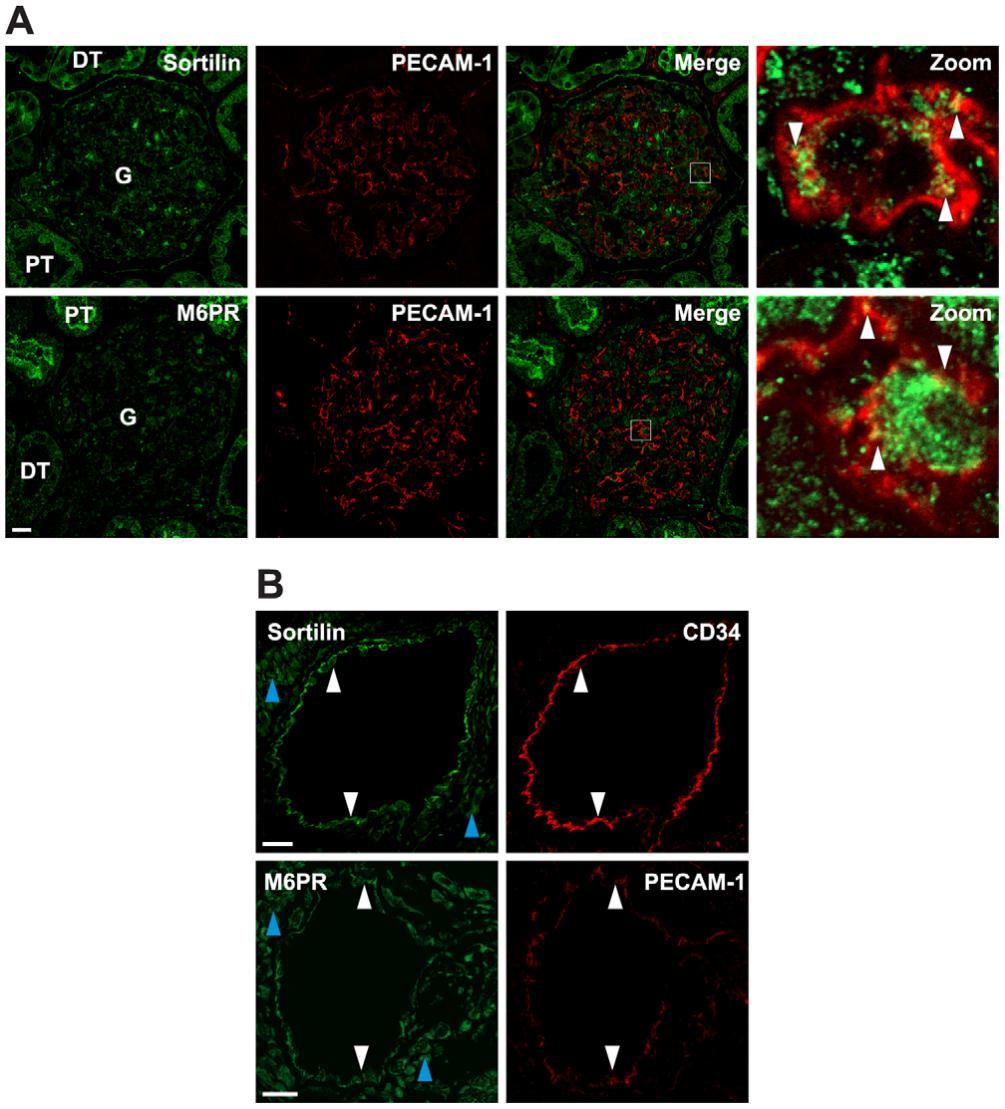
Expression of sortilin and M6PR in human GECs and in larger human renal vessel ECs. Dual immunofluorescence shows co-localization of sortilin and M6PR with PECAM-1 in human GECs in the glomeruli (G) as seen in sections from a normal human kidney. The respective merge images are shown in both low and high magnifications. Sortilin and M6PR are localized in the GECs and co-localizes with PECAM-1 to some extent as seen by the merged images. High-power views demonstrate that the receptors are localized in the GECs as indicated with white arrowheads. Sortilin and M6PR labeling of podocytes is also observed as previously shown in podocytes [15]. Scale bars, 20 µm. (B) Dual immunofluorescence show co-localization of sortilin and M6PR with PECAM-1 and CD34 (EC cell surface markers), respectively. The receptors are localized in the ECs of the larger renal vessels as indicated with white arrowheads. Staining of sortilin and M6PR is also seen in SMCs (blue arrowheads). Scale bars, 25 µm.

#### Localization of proteins in GECs

Coverslips with GECs were fixed with 2% paraformaldehyde, 4% sucrose in PBS for 10 min and with or without permeabilization with 0.3% Triton X-100 (Sigma Aldrich) in PBS for 10 min. Nonspecific binding sites were blocked with 4% FBS +0.1% Tween 20 (Sigma Aldrich) in PBS for 60 min and the cells were incubated with primary antibodies over night at 4°C. After this, they were incubated with Alexa Fluor conjugated antibodies for 1 h at room temperature, and nuclei-stained with DAPI. For co-localization of M6PR and sortilin with α-Gal A, GECs were incubated with 400 nM Alexa Fluor 546-labeled α-Gal A at 37°C for 1 h prior to localization of the proteins.

All coverslips were mounted on glass slides in DAKO Fluorescent Medium. Staining was analyzed by Zeiss confocal laser scanning microscope. Controls for nonspecific binding were performed with nonspecific serum and IgG fractions.

### Protein extraction

Cell proteins were extracted by addition of modified RIPA buffer (R-0278; Sigma Aldrich) containing complete protease inhibitors (Roche Diagnostics, Mannheim, DE; 1 tablet per 50 ml of solution) at 4°C. The suspension was centrifuged at 14,000x g for 15 min at 4°C, and the supernatant containing cellular protein was collected. Total protein was quantified using Pierce BCA Protein Assay kit (Pierce, Rockford, IL, USA) according to the manufacturer.

### Affinity purification of α-Gal A binding proteins

Purified recombinant α-Gal A was covalently coupled using the AminoLink Plus Immobilization Kit (Pierce) following the guidelines from the manufacturer. Affinity purification was done as previously described [15]. Eluted Fractions were collected, subjected to SDS-PAGE, and stained by silver staining using SilverSNAP Stain Kit II (Pierce). Additional gels were blotted onto PVDF membranes for further protein identification by Western blotting.

### SDS-PAGE and Western blotting

Eluted α-Gal A binding protein fraction with the highest protein content and GECs lysates were loaded on the SDS-PAGE gels. The samples were mixed with NuPAGE LDS sample buffer (Invitrogen; Carlsbad, CA, USA) with 2.5% SDS, and the proteins were separated by SDS-PAGE and electrophoretically transferred to PVDF membranes (Millipore Corporation; Bedford, MA, USA) for Western blot. Blots were blocked with 5% milk in PBS-T (80 mM Na_2_HPO_4_, 20 mM NaH_2_PO_4_, 100 mM NaCl, and 0.1% Tween 20 [pH 7.5]) for 1 h and incubated overnight at 4°C with primary antibody in PBS-T with 1% BSA. After washing in PBS-T, the blots were incubated for 1 h with horseradish peroxidase–conjugated secondary antibody (DAKO). After a final wash, antibody binding was visualized using ECL system (Amersham International, Bucks, UK).

### ^125^I-labeled α-Gal A uptake by GECs

Recombinant α-Gal A was iodinated with the use of the chloramine-T method [19], as previously described [15]. Human GECs were incubated with approximately 20 nM ^125^I-α-Gal A for different times. GECs were also incubated with different concentrations of ^125^I-α-Gal A for 12 h at 37°C as indicated. For uptake and inhibition studies, human GECs were incubated with approximately 20 nM ^125^I-α-Gal A in the presence or absence of 100 µM M6P, 10 µM RAP, 1 µM unlabeled α-Gal A, and both M6P/RAP at 37°C for 12 h. Incubation with ^125^I-α-Gal A was carried out in serum-free medium (EGM2-MV) containing 0.1% BSA (Sigma Aldrich), and stopped by aspiration of the medium. Cells were washed in PBS and collected by trypsinization with 150 µl of Trypsin-EDTA. The samples were counted in a γ-counter (Cobra 5003, Packard, Meriden, CT, USA). Degradation was measured by precipitation of the medium in 10% TCA and the TCA-soluble fraction, defined as the degraded fraction. To correct for liberation *per se* of iodine from ^125^I-α-Gal A during the experiment, the medium in control wells was incubated without cells. Degradation was calculated as the TCA-soluble fraction in the incubation medium minus the TCA-soluble fraction in the medium from control wells. Cell-associated ^125^I-α-Gal A was measured by counting the cells after trypsinization. Total uptake was defined as the sum of degraded and cell-associated fraction divided by the total amount of tracer added in each well. The medium from control wells contained a TCA-soluble fraction of approximately 5%.

### Surface plasmon resonance

For the surface plasmon resonance (SPR) analyses, BIAcore sensor chips (type CM5; BIAcore, Uppsala, SE) were activated as previously described [16]. Recombinant human soluble M6PR and sortilin were immobilized to densities 72 to 75 fmol/mm^2^. The samples in flow buffer were injected and the binding data was analyzed using the BIAevaluation program as previously described [15].

### Statistical analysis

The results of ^125^I- α-Gal A uptake experiments are presented as means ± SD, and the t test was used to test for significant differences. P values <0.05 were considered significant.

## Results

### Kidney endothelial cells take up recombinant α-Gal A

Using immunohistochemistry, we have previously shown that in a classic male Fabry patient, α-Gal is not detectable [15]. Here, we show for the first time that 2 hours after infusion of α-Gal A the enzyme is detectable in GECs of a renal biopsy from a Fabry patient (Figure 1A) and colocalizes with the endothelial cell surface marker CD34 as demonstrated by double-immunofluorescence staining. Immunoperoxidase staining also show that α-Gal A is detectable in endothelial cells (ECs) of larger renal vessels in the same Fabry patient after ERT (Figure 1B).

In conditionally immortalized human GECs [18], recombinant α-Gal A is taken up in a time- dependent manner (Figure 1C). The endocytosed enzyme was localized to the lysosomes as confirmed by co-localization of Alexa-Fluor 546 conjugated α-Gal A with LysoTracker-green (Figure 1D).

### Isolation and identification of M6PR and sortilin as α-Gal A-interacting proteins in GECs

Solubilized extract from cultured GECs was passed over either a recombinant α-Gal A coupled resin or a control resin. Comparison of the eluates from the two columns revealed that two protein bands with apparent masses of 250 and 100 kDa were present in the fractions eluted from the α-Gal A resin but not the control resin (Figure 2A). To determine the identity of the proteins, the eluted fractions were run on SDS-PAGE gel followed by immunoblotting. The proteins were identified as M6PR and sortilin using the corresponding antibodies (Figure 2B).

### Cultured human GECs express sortilin and M6PR

Immunofluorescence studies demonstrated that both sortilin and M6PR were expressed at the cell surface of cultured human GECs (Figure 3A). The expression of sortilin and M6PR was also demonstrated in permeabilized GECs showing prominent intracellular labeling (Figure 3B), as would be expected for sorting receptors. The phenotype of GECs was characterized by using a specific endothelial antibody-marker to PECAM-1 under permeabilized conditions (Figure 3C). Recombinant α-Gal A taken up by GECs co-localize with both sortilin and M6PR in intracellular compartments after 60 min (Figure 3D).

### Sortilin and M6PR mediate specific uptake of α-Gal A in human GECs

The endocytic activity of M6PR and sortilin expressed by cultured human GECs was investigated by their ability to mediate binding, internalization, and degradation of α-Gal A. ^125^I-α-Gal A was bound, internalized, and degraded at 37°C (Figure 4A–4E). The human GECs took up ^125^I-α-Gal A in both a time- and dose-dependent manner (Figure 4A–4D). The uptake is shown as total uptake including degradation products found in the medium (Figure 4A and 4C) and cell associated uptake (Figure 4B and 4D). Addition of M6P to the medium inhibited the α-Gal A uptake by approximately 59% after 12 h and receptor associated protein (RAP), which is well-known to inhibit the binding/uptake of sortilin ligands [20], inhibited 29% after 12 h (Figure 4E). Addition of excess unlabeled α-Gal A inhibited the uptake 53% after 12 h (Figure 4E). Finally, the combined inhibition by M6P and RAP was 76% after 12 h (Figure 4E).

### Recombinant α-Gal A binds to human M6PR with very high affinity

Surface plasmon resonance (SPR) analysis has already shown that α-Gal A binds to sortilin with a K_d_ of 400 nM. SPR analyses have also shown that recombinant α-Gal A binds to bovine M6PR, here we show that α-Gal A binds to immobilized human M6PR (41-1365aa) containing the two distinct M6P-binding sites (repeating segments 3 and 9) [21] (Figure 5A). Using the BIAevaluation program, K_d_ was estimated to 0.2 nM. We also examined if M6P inhibited the binding of α-Gal A to M6PR. At 50 µM, M6P markedly inhibited (∼75%) the binding of α-Gal A to M6PR (Figure 5B). We also tested the binding of α-Gal A to immobilized M6PR (1510-2108aa), which contains the single IGF-II-binding site (segment 11) [21], but no significant binding was observed (data not shown). Furthermore, M6PR (41-1365aa) binds to immobilized sortilin, and this binding prevents the binding of α-Gal A to sortilin (Figure 5C) demonstrating that the binding of M6PR (41-1365aa) to sortilin does not enhance α-Gal A binding to the M6PR-sortilin receptor complex, as the binding is inhibited.

### Expression of sortilin and M6PR in human kidney endothelial cells

Immunohistochemistry revealed that sortilin and M6PR were expressed in the ECs of normal human kidney (Figure 6A and 6B). The antibodies have previously been shown to be specific, as established by preabsorption of the two antibodies with their respective antigens [15]. Immunofluorescence shows localization of sortilin and M6PR in GECs as validated by co-localization with PECAM-1 (Figure 6A). Immunofluorescence staining also showed expression of sortilin and M6PR in the ECs of the larger renal vessels (Figure 6B) as validated by co-localization with CD34 or PECAM-1. Sortilin and M6PR were also expressed in SMCs of the vasculature in the human kidney (Figure 6B).

## Discussion

The present study was carried out to identify the mechanism of α-Gal A uptake in human GECs. We found that infused recombinant α-Gal A accumulated in the GECs and the uptake was targeted to the lysosomes by the cation-independent M6PR and sortilin. To our knowledge, none of these findings have been reported previously in human GECs.

Vascular endothelial cells are one of the severely affected cells in Fabry disease. [1] The mechanism by which this accumulation causes symptoms of disease is still unknown. Enhanced vascular thrombosis has been demonstrated [22], [23], [24], as well as altered endothelial-dependent vascular reactivity [25], [26], [27], increased markers of endothelial activation and endothelial microparticles [28], [29], and increased production of reactive oxygen species [30], [31], [32] in patients with Fabry disease and in Fabry mouse models. Endothelial dysfunction has been shown to lead to prominent vasculopathy in patients with Fabry disease similar to that of diabetic nephropathy [1], [33]. The accumulation of GL-3 in GECs, which together with the glomerular basement membrane and podocytes, play a pivotal role in glomerular permeability and selectivity, may predispose to albuminuria in Fabry disease through direct effects on GECs.

ERT with recombinant α-Gal A was reported to clear the GL-3 deposits in a variety of ECs in patients with Fabry disease [1], [7], [8], [9], [34], [35]. However, the mechanism of GL-3 clearance in the GECs and renal ECs has not been elucidated. Immunohistochemical localization of recombinant α-Gal A showed that after 2 h of ERT, the recombinant α-Gal A was taken up by GECs and renal ECs in a Fabry disease patient, but also in cultured human GECs.

We identified sortilin and M6PR as responsible for the α-Gal A uptake in GECs. Immunofluorescence analysis demonstrated for the first time that sortilin and M6PR were expressed in the GECs as verified by co-localization with PECAM-1 and CD34. Furthermore, immunofluorescence and Western blot studies also showed that the expression of these receptors could be detected in cultured human GECs. We have previously described that α-Gal A bound to sortilin with high affinity and was involved in the receptor-mediated uptake of recombinant α-Gal A in human podocytes and HEK293 cells transfected with sortilin [15]. In this study we show for the first time that α-Gal A binds to the human M6PR through the M6P-binding sites with very high affinity, but does not bind to the IGFII-binding site on M6PR. Sortilin is a multifunctional lysosomal receptor belonging to the Vps10p domain receptor family and homologous to that of the M6PR [36], [37]. Thus, M6PR and sortilin are sorting receptors typically found in intracellular compartments of the trans-Golgi network and early endosomes and have roles in both endocytosis and intracellular trafficking of proteins [37], [38], [39], [40]. However, we cannot discard the possibility that other pathways may exist as well for the cellular uptake of α-Gal A in the GECs and renal ECs, probably some form of fluid phase endocytosis may take place.

Recombinant α-Gal A binds to M6PR with higher affinity than to sortilin. We have previously shown that α-Gal A binds to sortilin with a K_d_ of 400 nM. In this study we show that α-Gal A binds to M6PR with a higher affinity, K_d_ of 0.2 nM. Recombinant α-Gal A binds to sortilin on the Vps10p-domain, as the binding and uptake of α-Gal A by sortilin can be inhibited by neurotensin, RAP and sortilin propeptide [15]. This is further supported by a study that reveals the crystal structure of sortilin ectodomain in complex with neurotensin. Here authors show that neurotensin binds to sortilin in the tunnel of a ten-bladed β-propeller domain [41]. The M6PR/IGF-IIR contains three binding sites; two M6P-binding sites and one IGF-II [21]. Recombinant α-Gal A binds to the M6P-binding sites and not to the IGF-II binding site as shown by the BIAcore studies. This supports the suggestion that a high number of M6P moieties on α-Gal A [42] would improve the uptake and delivery of α-Gal A to different cells by the M6PR.

Identification of M6PR and sortilin in human GECs furthermore suggests that, under physiological conditions, these receptors could be involved in the uptake of constituents from the plasma. Finally, M6PR and sortilin may be involved in sorting and signaling in GECs [37]. The expression of sortilin in both GECs and podocytes suggest that sortilin may be involved in new signaling cascades important for communication between these closely associated cells.

The delivery of α-Gal A to other ECs such as the macrovascular ECs, which are phenotypically and functionally different from microvascular ECs [43], [44], has not been tested. The EC-types show great variability [45]. However, we have shown that sortilin and M6PR were highly expressed in both larger vessels and capillaries in the kidney. Thus the receptors may have similar functions in microvascular and macrovascular systems.

In conclusion, these data demonstrate that M6PR and sortilin were expressed in human GECs and mediated the uptake of recombinant α-Gal A. We also show that sortilin and M6PR were expressed in the ECs of the larger vessels of the kidney. Thus we provide evidence of an additional receptor, sortilin, to the general M6PR in ECs cells for the delivery of therapeutic recombinant α-Gal A in Fabry disease and potentially other lysosomal storage disorders.
